# Maternal Health in Gujarat, India: A Case Study

**DOI:** 10.3329/jhpn.v27i2.3366

**Published:** 2009-04

**Authors:** Dileep V. Mavalankar, Kranti S. Vora, K.V. Ramani, Parvathy Raman, Bharati Sharma, Mudita Upadhyaya

**Affiliations:** Centre for Management of Health Services, Indian Institute of Management, Vastrapur, Ahmedabad 380015, India

**Keywords:** Health services, Management capacity, Maternal health, Maternal mortality, Morbidity, India

## Abstract

Gujarat state of India has come a long way in improving the health indicators since independence, but progress in reducing maternal mortality has been slow and largely unmeasured or documented. This case study identified several challenges for reducing the maternal mortality ratio, including lack of the managerial capacity, shortage of skilled human resources, non-availability of blood in rural areas, and infrastructural and supply bottlenecks. The Gujarat Government has taken several initiatives to improve maternal health services, such as partnership with private obstetricians to provide delivery care to poor women, a relatively-short training of medical officers and nurses to provide emergency obstetric care (EmOC), and an improved emergency transport system. However, several challenges still remain. Recommendations are made for expanding the management capacity for maternal health, operationalization of health facilities, and ensuring EmOC on 24/7 (24 hours a day, seven days a week) basis by posting nurse-midwives and trained medical officers for skilled care, ensuring availability of blood, and improving the registration and auditing of all maternal deaths. However, all these interventions can only take place if there are substantially-increased political will and social awareness.

## INTRODUCTION

Gujarat state, situated on the west coast of India, accounts for 6% of the area of the country and 5% (51 million) of the population of India making it rank tenth in the country (1). The decadal population growth rate (1991-2001) of the state has been 22.6%, which is higher than that of India (21.5%) (2).

It is one of the most urbanized states in India, with 37% urban population, perhaps because agriculture is highly unstable, leaving small farmers and farm-labourers in poverty. The eastern tribal belt and the northern dry region remain underdeveloped parts of the state. Overall, Gujarat has 25 districts subdivided into 226 blocks, 18,618 villages, and 242 towns (3). Table 1 provides a comparison of India and Gujarat on important demographic and health indicators.

**Table 1.** Demographic and health indicators of Gujarat and India

Indicator	Gujarat	India
Population (million) (census 2001)	51	1,028
Decadal growth rate (1991-2001)	22.6	21.5
Population density per sq km (2001)	258	324
Birth rate (2006)	23.5	23.5
Death rate (2006)	7.3	7.5
Total fertility rate (2005)	2.9	3.2
Age (years) of effective marriage (2005)	20.3	20.2
Literacy rate: total (2001)	69.9	65.3
Male	80.5	75.3
Female	58.6	54.1
Sex ratio (no. of females per 1,000 males)	920	933
Life expectancy at birth—females (2005)	69.0	66.1
Infant mortality rate (2006)	53	57
Child mortality rate (2005)	16	17.3
Maternal mortality ratio (2003)	172	301

Source of data: India. Ministry of Health and Family Welfare. Family welfare statistics in India–2006: Table A-7 (2)

Table 1 shows that Gujarat is doing better than the national average for most demographic and health indicators. It has a higher literacy rate, lower total fertility rate (TFR), higher life expectancy at birth for women, and less population per sq km. Its infant mortality rate (IMR) is 53 versus the national rate of 58 (4), and the child mortality rate (CMR) is 16 vs 17.3 respectively. A higher IMR among girls was observed compared to boys in urban Gujarat. As per the maternal mortality study conducted by the Registrar General of India, based on the Sample Registration System (SRS), the maternal mortality ratio (MMR) for Gujarat was 172 compared to 301 for India for 2001-2003 (5). Gujarat has a 14.8% population of backward tribes, called Scheduled Tribes and 7.1% backward castes, called Scheduled Castes (6).

### Status of women

Female literacy is low (59%) compared to male literacy (81%) in Gujarat. Although Gujarat has a higher female literacy rate than the national average, the sex ratio is more adverse for females. This indicates son preference and low status of women in society. Ahmedabad, Mehsana, Gandhinagar, and Rajkot districts with a female literacy rate of above 60% have low sex ratios, but Dahod, Dangs, and Narmada with hardly 35-40% of female literacy have better sex ratios (7). The age of effective marriage for women is the same for India and Gujarat at 20 years.

The purpose of the case study was to analyze the present situation in Gujarat regarding maternal health compared to that in India and to understand the constraints to, and possibilities of, improving maternal healthcare in the state.

## MATERIALS AND METHODS

This paper is based on the case-study methodology used in management studies. Case studies generally document real situations of organizations from multiple perspectives and include a description of the organization, key data in terms of inputs, process, outputs, and perspectives of key individuals obtained through interviews. As opposed to focused research, there may not be a clear hypothesis; however, the problems faced by the organizations and some analyses are provided.

This case study analyzes how the current situation of the healthcare system affects the performance indicators of maternal health. Past efforts in the maternal health area were analyzed to know their impact and challenges. New initiatives were studied to understand their potential. The case study is based on a review of literature (published and unpublished reports of government and non-governmental agencies), collection of secondary data from the management information systems of the national, state and district levels, interviews with stakeholders, study of key institutional processes, roles and authority of key actors, and organizational structure and function. Quantitative data on maternal health were taken from the three National Family Health Surveys (NFHSs) (1992-2006) (8-11) and District Level Household Survey (DLHS) (2002-2003) (12). Information regarding health infrastructure and human resources was collected from a nationwide facility survey (2004-2005) which included Gujarat. Data and information are also obtained from official website of the health and family welfare department of the Gujarat Government.

### Sources of data

Sources of data used by the Department of Health are (a) routine statistics from the health management information systems (HMISs), (b) population-based surveys, (c) facility surveys, and (d) special studies/evaluation by external agencies. The HMIS routinely collects information on maternal health services. However, there are issues of over-reporting by staff, non-reporting, under-reporting, variable coverage, delays in receipt of reports, and lack of data verification (13). As in the rest of India, Gujarat also has an SRS—an independent system—which collects data on vital statistics regularly and publishes reports.

Among the sources of data, the NFHSs are considered to be the most reliable as the sample size is large, and an independent agency conducts the surveys. The DLHS also provide insights into the maternal health situation in Gujarat as they collect information on coverage of antenatal care, institutional delivery, skilled birth attendance, etc.

This study is limited by the inability to obtain reliable data on maternal mortality and morbidity in Gujarat. No independent systematic evaluations of the maternal health programme were carried out.

## MATERNAL HEALTH INDICATORS

According to a special study on maternal mortality conducted by the Registrar General of India, based on the SRS (which was originally meant to calculate crude birth rate, crude death rate, and IMR), the MMR of Gujarat has fluctuated from 46 for 1997-1998 (95% confidence interval [CI] 90-74) to 202 in 1999-2000 (95% CI 141-262) and 172 in 2001 and 2003 (95% CI 116-228), giving a simple average of 140 between 1997 and 2003. This is comparable with other southern states of India (6). These estimates are based on a population of 216,000 and 21,000 births in Gujarat. While analyzing these studies, it was felt that these rates are underestimated as the actual MMR could be higher—in the range of 200-300. This increase is similar to that determined by the international maternal mortality working group for the MMR of India—approximately one and half times higher at around 450 from 301 estimated by the Registrar General of India (RGI) from a SRS survey is 2003 (14).

Table 2 shows the trend over time for some maternal care-use indicators based on the NFHS data. Gujarat has progressed, although slowly, towards the use of better maternal healthcare in the last decade. The coverage of antenatal care (3 visits) is only 65%, and the incidence of anaemia among pregnant women has increased in the last five years. At present, more than half of the pregnant women in Gujarat are anaemic, despite programme emphasis for prophylaxis of anaemia and schemes to improve their nutrition. Results of a detailed analysis of 14% of women who did not receive any antenatal check-up in the NFHS-2 showed that these women were mostly high-parity rural women, from scheduled tribes, illiterate, and women with a low-standard of living (9).

**Table 2.** Maternal health indicators in Gujarat

Indicator	NFHS-1 (1992-1993) (%)	NFHS-2 (1998-1999) (%)	NFHS-3 (2005-2006) (%)
Pregnant women with anaemia	NA	47	61
Three antenatal check-ups	61	61	65
Institutional deliveries	36	46	55
Deliveries conducted by health personnel	43	53	65
Mothers received postnatal care within 2 days of delivery	NA	NA	54

Source of data: National Family Health Survey 3 (8)

NA=Not available; NFHS=National Family Health Survey

As per the NFHS data, institutional deliveries have increased to 55% in 2005 from 36% in 1992. According to the NFHS-2 (1998-1999), the private sector conducted about 32% of deliveries, and only 11% of institutional deliveries were conducted in public-sector facilities (10). The community and the first-tier workers (Auxiliary Nurse Midwife/Lady Health Visitor—ANM/LHV) conduct only 11% of deliveries. Doctors or staff nurses conduct the majority (52%) of deliveries in the public sector. The rate of caesarean section has increased from 3% in the NFHS-1 to 8.5% in the NFHS-2, again reflecting the predominance of the private sector in institutional deliveries (Table 3). As per the NFHS-3, *dais* conducted 32% of deliveries which is lower than (45%) in the NFHS-1.

**Table 3.** Comparison of performance indictors for maternal health services in Gujarat over time from the three NFHSs

Indicator	Gujarat		
	NFHS 1 1993	NFHS 2 1999	NFHS 3 2006
Coverage of antenatal services			
Tetanus toxoid injection (2 or more)	63	73	80.4
Completed 3 antenatal care visits	61	60	65
Received iron/folic acid tablets	69	78	82.4
Place of delivery			
Institutional deliveries	36	46	55
Domiciliary deliveries	64	54	45
Institutional deliveries			
Public	15	11	13.9
NGO/trust	NA	3	2.0
Private	20	32	36.8
Type of delivery			
Vaginal delivery	97	91.5	91.1
Caesarean section	3	8.5	8.9
Assistance during delivery†			
Doctor	29	37	52.0
ANM/nurse/midwife/LHV	14	16	11.0
Other health professionals	NA	--	0.3
*Dai* (TBA)	45	42	31.6
Other	12	4	5.1

Source of data: National Family Health Survey 2 (10) and National Family Health Survey 1 (11)

†If a respondent mentioned more than one attendant, only the most qualified attendant was considered; ANM=Auxiliary Nurse Midwife; LHV=Lady Health Visitor; NA=Not available; NFHS=National Family Health Survey; NGO=Non-governmental organization; TBA=Traditional birth attendant

Maternal education plays an important role in awareness of, and access to, healthcare. Table 4 shows the less use of maternal health services by illiterate women compared to those who have completed high school.

**Table 4.** Comparison of the use of maternal health services by educational status of mothers (NFHS 2)

Indicator	% of illiterate women	% of women who have completed high school
Any antenatal care	78	99
Institutional delivery	44	78
Delivery assisted by doctor/ANM/LHV/nurse	36	83
Postnatal care at any time	8	16

Source of data: National Family Health Survey 2 (10)

ANM=Auxiliary Nurse Midwife; LHV=Lady Health Visitor; NFHS=National Family Health Survey

## SERVICE-DELIVERY SYSTEM IN MATERNAL HEALTHCARE

Following the National Health Policy (NHP) of 1983, a ‘three-tier' healthcare system was established in Gujarat. Level-I is the Subcentre (SC) covering a population of 5,000 and Primary Health Care Centre (PHC) with six beds located at large villages catering to a population of about 30,000. Level-II is Community Health Centres/First Referral Units (CHCs/FRUs) with CHCs at 100,000 people and FRUs at 500,000 people, and level-III is the district hospital with 100-300 beds located at the district town. These facilities have increasing levels of staff and specialty and also facilities for advanced treatment (Table 5).

**Table 5.** Public-health facilities and providers by level and population coverage

Healthcare institution	Standard norm population	Actual population covered (2005)	No. in Gujarat (2006)	Level	Highest medical services provider
Medical college hospitals	5-8 million	NA	08	Apex	Super specialists
District hospital	2-3 Million	NA	25	III	Specialists, including obstetrician
First Referral Unit	3- 5,00,000	NA	22	II	Obstetrician
Community Health Centre	1- 3,00,000	116,694	272	II	Medical officer/specialists
Primary Healthcare Centre	20,000-30,000	29,664	1,072	I	Medical officer, staff nurse
Subcentre	3,000-5,000	4,364	7,274	I	Auxiliary Nurse Midwife, multipurpose worker (female)

Source of data: Rapid Household Survey Bulletin, March 2006 (15)

Although the healthcare-delivery system looks good on paper, it has many problems which affect service-delivery in maternal healthcare. The key problems are inadequate infrastructure and equipment, shortage of human resources, lack of supplies, and inadequate monitoring and supervision. These problems are discussed in details below.

## ORGANIZATIONAL STRUCTURE AND MANAGEMENT CAPACITY OF THE HEALTHCARE SYSTEM

### State level

**Fig. 1.**
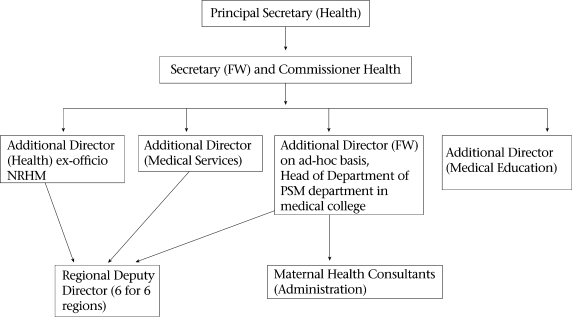
Organizational chart at state level for maternal health services, Gujarat

The Department of Health and Family Welfare (DHFW) is headed by a minister, under whom are two Additional Chief/Principal Secretaries (Fig. 1). Below them is the Commissioner of Health, who heads the technical wing of the DHFW, assisted by six Additional Directors. The major administrative divisions of the DHFW involved in the management of maternal health services are the Directorates of Rural Health, Medical Services, Medical Education and Research, Vital Statistics, and Family Welfare, and State Institute of Health and Family Welfare. All Additional Directors are doctors who do not necessarily have public-health or management qualifications.

FW=Family welfare; PSM=Preventive and social medicine; RCH=Reproductive and child health

Although the reduction in the MMR is a key health-policy objective, Gujarat does not have a separate director in charge of maternal health. The current Commissioner, who has training in public health, has made efforts to bring in technical officers—called consultants—to look after management of maternal and child-health services on ad-hoc basis. No additional permanent full-time positions have been created for maternal and child health in recent past.

The Additional Director, Health and Family Welfare, is also the Programme Director of Reproductive and Child Health (RCH) II programme, in charge of all components of RCH, including maternal health. At present, this position is occupied by a professor and head of preventive and social medicine from one of the medical colleges, holding dual charge and multiple responsibilities. As there are only three officers for maternal health and as they are responsible for many activities, they cannot do full justice to all maternal health strategies. For example, many activities to strengthen maternal health under the CSSM programme (1992-1996) and RCH programme (1997-2004) could not be implemented (16). These include operationalization of FRUs, ensuring essential obstetric care, operationalization of blood-storage units, implementation of evidence-based preventive practices, such as active management of the third stage of labour and partograph, and monitoring of maternal deaths and complications of child birth. Progress on these interventions has been very slow over the past 5-7 years, although some have picked up speed after the appointment of the two maternal health consultants in the recent past. From the two consultants for maternal health, only one is full-time who looks after the administrative aspects of maternal health. The other one is a professor of obstetrics from a teaching hospital who is responsible for coordinating training of skilled birth attendants (SBAs), training on EmOC, and operationalizing the FRUs. He comes to the Directorate five days a week and spends one day a week in the medical college. The limited management capacity has been a major hindrance to implementing the maternal health programmes.

### Regional level

**Fig. 2.**
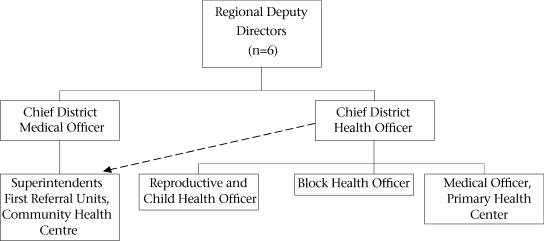
Organizational chart at district level for maternal health services, Gujarat

The Gujarat state is administratively divided into six health regions—each with 5-6 districts in a region—headed by a Regional Deputy Director (RDD) (Fig. 2). The regional directorates were set up in 1986 to decentralize authority and responsibility of managing day-to-day problems at health facilities, and policy-level decisions are taken at the state level. All government hospitals, including the FRUs and CHCs, now come under the RDD. The RDDs should monitor the functioning of the FRUs and district hospitals for EmOC, but it is not happening. The RDDs find themselves without adequate authority to perform the duties of managing health facilities efficiently.

### District level

To increase local control, the PHCs were handed over to the District Councils (DCs) in Gujarat in the 1960s. However, it has not yeilded desired results possibly because the elected representatives were unaware of the complexities of the health sector and were unwilling to enforce accountability. The state Department of Health has not been able to use the DCs for mobilizing communities and enforcing accountability. Our meetings with the DCs revealed that the State Government has neigther organized orientation programmes nor prepared any self-learning material on various health programmes for the DCs. It seemed that the district administration did not want elected represenataives to know about health programmes for fear that they would start interferring politically with various programme activities. This left the DCs unaware of various health schemes and their authority. For example, many members of the DCs did not know that the they can hire ANMs at the district level; they were requesting the states to do so.

A district has two hierarchial systems—one controlling PHCs providing mainly preventive MCH services and the other controlling CHCs and district hospitals providing curative services. Maternal health services at the PHC level are supervised by the RCH officer, working under the Chief District Health Officer (CDHO). Both are doctors with a Diploma in Public Health. Administrative and financial powers lie with the District Development Officer (from the non-technical Indian Administrative Services–IAS) leading to delays in decision-making and implementation of programmes at the field level.

Public Health Nurses (PHNs) supervise the LHVs and ANMs at the district level. There are two posts of PHNs; however, most districts have only one post filled. Recently, at the district level, a new unit—the District Programme Management Unit (DPMU)—has been set up under the RCH programme, staffed by a health management graduate and support staff. It is unclear what this unit will do for maternal health.

Curative services are monitored by the Chief District Medical Officer (CDMO), a doctor with postgraduate degree in medical or surgical specialty, in charge of all medical services in the district, including the CHCs and district hospital. One in five CHCs is designated as a FRU catering to 500,000 people and is supposed to provide comprehensive EmOC services. The activities of CDMO are monitored by the Additional Director, Medical Services, at the state level, not by the Additional Director, Family Welfare, which weakens the linkages of medical services with maternal health services.

### Subdistrict level

A medical officer, a staff nurse, and an LHV at the PHC are jointly responsible for providing antenatal care, conducting deliveries, providing basic EmOC and referral, postnatal care, and family-planning services in an area of 30,000 people. The ANMs are posted at the health subcentre covering a population of 5,000. They are responsible for house-visits to women, registering pregnant women, motivating them to obtain antenatal services, and institutional delivery. An ANM is supposed to attend deliveries at home, provide postnatal care, and refer women with complications. Beginning in 1966, with the target-oriented family-planning programme, programme priorities shifted from delivery care to family planning and immunization programme (1980s onwards). Both family planning and immunizations are primary preventive activities carried out by the ANMS and can be done on a periodic basis. Both these lead to neglect of delivery care by the ANMs and MOs.

## BLOOD-BANKING SERVICES IN GUJARAT AND ITS IMPACT ON MATERNAL HEALTH

Gujarat has 162 functioning blood banks for a population of 51 million or one blood bank per 313,000 people. This ratio of blood banks to population is worse in rural areas, as most blood banks are located in urban areas.

The Gujarat State AIDS Control Society (GSACS) is the controlling body for all blood banks. Their management capacity is, however, inadequate, and control over the blood banks is somewhat weak; for example, only 112 of the 162 blood banks provide an annual report to the GSACS. There is no full-time state-level officer to improve blood-transfusion services; however, there is a blood-safety officer in the GSACS whose role is to ensure safety of blood through ensuring proper testing.

Donation of blood has a long history in Gujarat beginning with the Red Cross setting up the voluntary blood-donation movement in Gujarat in the 1960s. The Gujarat State Council for Blood Transfusion was established in the 1990s for increasing the availability of safe blood and promotion of voluntary blood donation/use of blood components. Only 50 of the 162 licensed blood banks are allowed to conduct outdoor blood-donation drives in the community by the Gujarat blood-donation council.

Given the high level (61%) of anaemia in the state (Table 1), it would not be unrealistic to assume that 5% of the 1.2 million annual deliveries in Gujarat require blood transfusion. This translates into a total demand of 120,000 units of blood given if each woman needing blood is transfused with two units. This is about 25% of the total blood units collected in Gujarat. Haemorrhage is the major killer of mothers, and blood transfusion plays an important role in saving mothers. Access to blood is essential for any EmOC services, and looking at the present situation, it is clear that many mothers in Gujarat may die due to lack of access to blood.

The Government has made some efforts to improve the availability of blood in rural areas by starting to establish blood-storage units. Unfortunately, the pace of setting up blood-storage units has been quite slow. As per the norms, 41 more blood-storage facilities are required (17); only 15 such units have been made operational till 2007, although the policy for blood-storage units was promulgated in 2001.

Blood-bank services in Gujarat though good are plagued by shortcomings. These are discussed in detail in a separate paper on blood banking in this issue of the Journal (18).

## EMERGENCY TRANSPORT AND COMMUNICATION

Gujarat has not developed a systematic plan to improve the communication and transportation system for EmOC. Ambulances have been bought on an ad-hoc basis as and when budgets are available. Similarly, telephones were also installed without much long-term planning or specified protocol for using them in emergencies. However, with rapid expansion of telecommunication, almost 100% of the district hospitals, CHCs, and FRUs now have telephones; however, only about 60-70% of the FRUs and CHCs have functional vehicles as per the facility survey of 2003 (12).

In 2007, the Emergency Medical Research Institute, an NGO, has set up a network of high-tech ambulances with wireless communication with a central call centre that can be reached by a single telephone number. Currently, these services are available across Gujarat, covering all 26 districts and 50 million population with a fleet of over 400 ambulances attending to over 1,800 emergencies per day (19).

## CHALLENGES IN MATERNAL HEALTH-SERVICE DELIVERY

The Central Ministry of Health and Family Welfare has taken stock of past programmes and formulated the RCH II (2005-2009) and National Rural Health Mission (NRHM), an all-encompassing strategy to bring together all health-related programmes in the country. This has resulted in maternal health services in Gujarat being refocused as a priority under the RCH II and NRHM programmes. Despite better understanding of effective strategies to improve maternal health and to reduce maternal mortality in Gujarat, the health system continues to face challenges in providing maternal health services. These are discussed below.

### Registration of maternal deaths and maternal death audit

The estimates of maternal mortality in Gujarat are primarily based on the SRS which covers small sentinel populations; hence, validity of data is an issue. For example, the estimate of MMR for Gujarat based on the SRS data for 1998 is 28 (as per the SRS 2000), and as per the SRS 2006, the MMR is 173. The vital registration system in Gujarat is also lacking, and substantial numbers of maternal deaths are not registered; for example, in 2005, of an estimated 3,000-4,000 maternal deaths, only 1,333 were registered. Although a maternal death-audit system was recently started, it is at a nascent stage. No officer is solely responsible for the registration of maternal deaths, analysis of the audits, and reporting. Without correct estimates and a good maternal death-registration system, it is difficult to gauge the severity of the problem and take corrective steps or to know the effectiveness of measures taken to improve maternal health services.

### Infrastructure for maternal health services

Standards of health infrastructure, equipment, logistical and administrative support differ according to the level of health facility. Higher-level facilities, e.g. medical colleges and district hospital, tend to have more infrastructure, equipment, and trained staff than do the CHCs and Primary Health Centres (PHCs) and subcentres. The general maintenance of the facilities influences the quality of services. For maternal health, especially EmOC, it is imperative to have staff on a 24/7 basis and an accessible infrastructure which includes a quality labour room and operation theatre facilities. Lack of infrastructure, such as quarters for staff, clean water, electricity, and good schools at the subcentre, PHC and CHC village, makes it difficult for staff to live with their families in rural areas. In many cases, the location of the facility is isolated at the outskirts of the village, with no approach-road, making it inaccessible in the monsoon. Government buildings have poor quality of construction, and maintenance is difficult due to lack of appropriate policy, money, priority, and cumbersome procedures, thus affecting the quality of services. Although, as per the DLHS-2 (2002-2004), 89% of 1,072 PHCs had adequate infrastructure (12), they are not properly maintained. The quality of infrastructure deteriorated over time; so, it is difficult to provide maternal health services in absence of adequate infrastructure. Fortunately, some improvement in infrastructure has taken place following the 2001 earthquake and funds coming under the NRHM since 2006.

### Human resources for maternal healthcare

The most crucial challenge faced by the state is ensuring skilled birth attendance and availability of trained personnel at the FRUs for comprehensive EmOC.

All past programmes, starting with the CSSM in 1992, had a component of training the general doctors for 6-12 months on EmOC or on anaesthesia. Due to lack of focus, poor training facilities, objections from professional bodies, legal problems, and posting policies, not much progress has been achieved. Recently, training of MBBS doctors has started and is going well but problems of posting policies, absence of supervision, accountability, and lack of infrastructure and supply support are still not addressed.

The ANM is the first-level contact for the community in the primary healthcare system at the village level. She is the main provider of antenatal, delivery and postnatal care services in rural areas. Yet, nearly 22% of posts of ANM are vacant in Gujarat, although it is difficult to estimate in the absence of accurate data (Table 6). These vacancies are caused by very weak human-resource planning and management in the state Department of Health. For example, forecasting required of health staff, including ANMs, has not been done regularly. Also, there is a little coordination between the training schools which train ANMs and the recruitment process at the district and state levels which can be long and politically influenced. In remote and difficult districts, adequate numbers of qualified candidates may not be available for ANMs.

**Table 6.** Human resources available in public-health infrastructure of Gujarat, 2006

Public-health worker	Sanctioned	Posted	Shortfall	% of shortfall
Multipurpose worker (female)/ANM at Subcentres and PHCs	8,346	6508	1838	22
Health Assistant (female)/LHV at PHCs	1,072	862	210	20
Health Assistant (male) at PHCs	1,072	616	456	43
Doctor at PHCs	1,072	907	165	15
Obstetricians and gynaecologists at CHCs	273	7	266	97
Paediatricians at CHCs	273	6	267	98
Radiographers	273	110	163	63
Pharmacist	1,345	797	548	41
Laboratory Technicians	1,345	865	480	36
Nurse/midwife	2,983	1,585	1,398	47

Source of data: Rapid Household Survey Bulletin, March 2006 (15)

ANM=Auxiliary Nurse Midwife; CHCs=Community Health Centres; LHV=Lady Health Visitor; PHCs=Primary Health Centres

Even if recruited, most ANMs do not live in the villages they are posted to but prefer living in a larger town or in a city that may be several kilometres away. The time available for service-delivery in the community is, thus, limited, and substantial time is spent in commuting. Other reasons for not staying at the place of posting are complex and multiple, such as lack of adequate housing facility, lack of safety and security, poor skills, and confidence to conduct deliveries, and a sociopolitical milieu which encourages lack of accountability. The ANMs not staying at their post cannot provide delivery care due to the unpredictably of childbirth. The TBAs, therefore, continue with conducting deliveries. Although this is a very important issue, the Government does not monitor the parameter of ANM's place of stay.

As described in the paper on maternal health situation in India in this issue of the Journal, antenatal care, delivery care, and postnatal care provided by ANMs are not monitored as strictly as immunization and family-planning activities because of the high priority for the latter programmes (16). The ANMs were supposed to be primarily community-based midwives but changes in programme focus from maternal care to family planning and immunization led to the shortening of training of ANMs in midwifery. The role of ANMs has essentially changed from provider of comprehensive maternal health services, including childbirth, to selected preventive services (immunization and family planning) provider.

The staff nurses and LHVs at the PHCs and CHCs are not adequately skilled to provide delivery care. In many places, staff nurses and LHVs did not also stay at the PHCs for reasons similar to those described for ANMs above, and hence, they are not available for 24 hours for delivery care. Medical officers posted at the PHCs are not skilled for delivery care and basic EmOC.

There is also a severe dearth of specialists in the public-health system. In Gujarat, there are no sanctioned posts for specialists for providing comprehensive EmOC functions, such as obstetricians, anaesthetists, or paediatricians, in the CHCs. Even where there is a sanctioned post of specialist, vacancy is extremely high (about 97%) (Table 6). These high rates of vacancies of specialists' posts are due to many reasons, prominent among them being that government doctors are not allowed to do private practice and working conditions in rural areas are difficult. The Government faces difficulties in disciplining employees due to strong employee unions and political interference. This leads to higher rates of absenteeism and avoidance of being transferred to remote areas. Most FRUs are non-functional due to lack of availability of specialists.

The management information system for human resources is also very weak. Data on filled-up/vacant posts are not updated regularly, and there is no monitoring of whether a posted healthcare provider stays at the place of posting.

## SOCIOPOLITICAL ENVIRONMENT AND CHANGE IN PROGRAMME PRIORITIES

Over time, maternal health services of the public-health system have changed from comprehensive care to only primary preventive care unlinked to referral services required to manage complications. Emphasis has shifted more to antenatal care than delivery care along with postnatal care. Indicators on deliveries conducted, use of postnatal care, and use of other maternal health services, although monitored, get less priority compared to familyplanning and immunization indicators. Even in the NRHM, the annual monitoring sheet does not have important maternal health indicators.

## LIMITED NUMBER OF MANAGERS AND MANAGEMENT CAPACITY

In a large state, like Gujarat, with its 1.2 million births per year, maternal health programming needs substantial management capacity to effectively plan, implement, and monitor various strategies to reduce the MMR and provide quality care to all mothers and newborns. The person in charge should have public-health and management skills besides the technical knowledge about maternal health. The key problem in the Commissionerate at the state level is that there are only two officers at the top level responsible for maternal health for all of Gujarat. Yet, none of the consultants is required to have a public-health background or management training because the recruitment/promotion rules do not specify such requirements. This could be due to lack of appreciation of the importance of management and public-health training and/or professional rivalries and the superior hierarchical position of Obstetricians and Surgeons in the health systems. Because of the prolonged financial crunch in the State Government and the perception that the government bureaucracy is bloated, the finance department is very reluctant to allow expansion of permanent positions and hire new staff. The procedure to create new top-level positions may be time-consuming and complicated. Yet, the main problem with contractual staff and consultants is that they are not permanent and could leave the department or revert back to their original positions, thus, making programme improvements less sustainable. Short-term consultants may not develop ownership of the programme and long-term vision for the development of maternal health in the state.

The limited management capacity at all levels is one of the major reasons for slow progress in maternal health in Gujarat despite ambitious programmes, such as RCH and CSSM. There is no permanent full-time post of maternal health officer or midwifery officer at the regional, district or subdistrict levels. At the district level also, only one officer is responsible for all reproductive health services, of which maternal health is one component. Administrative powers lie with an IAS officer and not with the district-level public-health officers due to lack of decentralization of authority to officers other than IAS; this makes district-level management difficult. At all levels, officers involved in the implementation and monitoring of maternal health services have other responsibilities, in addition to maternal health and, hence, are overburdened and cannot devote full attention to maternal health (20). In the absence of adequate management capacity, the maternal health programme is not well-planned, executed, or monitored, which results in poor quality of care, delays, non-implementation of evidence-based practices and under-use of services. This is not to disregard what has been achieved for the last few years by the hard work and untiring efforts of the Commissioner, maternal health consultant, and the team. However, we want to highlight the fact that, with a larger management team, much more could have been achieved.

To address this issue of the management capacity, the State Government has started creating ad-hoc posts of District Programme Coordinators, who are health-management graduates not necessarily medically qualified or trained in public health. There has also been an initiative to establish block health offices to improve the administrative processes and monitoring in the blocks (about 100 villages and 4 PHCs). Senior medical officers without training in public health or management man the block health offices. These two efforts have improved the management and monitoring processes to some extent.

## NEW INITIATIVES FOR IMPROVING MATERNAL HEALTH IN GUJARAT

### Chiranjeevi Yojana

The Government of Gujarat initiated a scheme—called the ‘Chiranjeevi Yojana' (in Gujarati, ‘Chiranjeevi' means long life and ‘Yojana' means scheme)—aimed at encouraging institutional deliveries and establishing a model of public-private partnership to reduce maternal mortality. Launched in December 2005, it began as a one-year pilot project in five backward districts of Gujarat. Since January 2007, the Gujarat Government decided to implement the scheme throughout Gujarat; the Central Government is also recommending the scheme to other states to improve institutional deliveries.

The Chiranjeevi Yojana focuses on providing free delivery care to women below poverty-line (BPL) through a private obstetrician. The Government has selected and empanelled willing private obstetricians owning small hospitals. The Government of Gujarat, after conducting various discussions, decided to pay a fixed predetermined amount (US$ 4,487) for 100 deliveries to each private obstetrician. This package of 100 deliveries includes both normal and complicated deliveries as per a fixed estimated proportion of complications in rural populations. Such a fixed payment schedule for 100 deliveries eliminates the financial incentive to do more caesarian sections and medical interventions than necessary. The benefits of the package also include free food and medicines after delivery for the woman and reimbursement of transport cost (US$ 1.25) for accompanying members of the family. The scheme is implemented in steps, including raising awareness, involvement of the community, selection of provider(s), and management of contract with regular payment to providers. Various officials are assigned specific roles in the scheme from the state, district, block, PHC to subcentre. The beneficiary has to present either a BPL card or pre-specified certificate of poverty from a Panchayat head or Medical Officer to avail of delivery services free of charge from the empanelled specialist. The ANM provides the list of empanelled private specialists in their area to poor women so that they can select their own doctor. The scheme has been quite successful. As of February 2008, 852 of 2,000 available private specialists have joined the scheme and conducted 1,65,278 deliveries. Of these, 10,278 (6.21%) were caesarean sections, and 11,118 (6.7%) were complicated deliveries (21).

A study carried out in 2007 by interviewing a sample of the Chiranjeevi Scheme beneficiaries and a sample of potential beneficiaries who did not use the scheme (controls) in one district found substantial reduction in the delivery expenditure by poor beneficiaries of the scheme compared to controls, although the poor still had to pay some money for delivery. The leakage of benefits to the non-poor people is very limited. Most beneficiaries were satisfied and quite happy with the scheme. However, there were some issues relating to clients, providers, and the health system which need to be considered before scaling up this scheme. Providers were not clear about the details of the scheme and were not happy with the remuneration. Some have withdrawn from the scheme after a while. There are also issues in obtaining the BPL card and awareness about the scheme (see the paper on the Chiranjeevi Scheme in this issue of the Journal) (22).

The scheme also has some unintended negative outcomes. For example, deliveries conducted at the government facilities have declined. The ANMs and medical officers were more involved in promoting the scheme than conducting deliveries themselves. Overall, the Chiranjeevi Yojana is a good example of the public-private partnership to provide delivery care to the rural poor but it needs further evaluation and fine-tuning.

### Training of MBBS doctors on emergency obstetric care

India has about 20,000 obstetricians mostly in the private sector who are based in cities. A very few specialists work in the public sector, and they are mostly at district hospitals. Gujarat started basic EmOC training for general doctors and staff nurses in 2003-2004 to improve access to skilled birth attendance in rural areas. An initial two-week basic training was started in district hospitals in five backward districts and then implemented in all the districts. At present, monitoring and selection of trainees are done at the regional level. All the CHCs are expected to be covered by 2008. Once all the CHCs are covered, the PHCs where deliveries take place would be given priority for training medical officers. By March 2008, about 600 medical officers and 1,200 staff nurses have been trained on basic EmOC.

In 2005, the Obstetrics and Gynecology Association of India (FOGSI) started an initiative, along with the Government of Gujarat, to train general doctors (Medical Officers with MBBS degree) in comprehensive EmOC. The design of the course and technical backstopping was done by JHPIEGO and the AMDD project. Master trainers from teaching hospitals were trained at the Christian Medical College, Vellore. The training is competency-based with the help of models and hands-on training. Six weeks of this 16-week comprehensive EmOC training cover theoretical training at the teaching hospital and 10 weeks in district hospital for practical training. Under this training, Medical Officers are trained for caesarean section, in addition to basic training. The training has been successfully carried out for the last 18 months with two batches per year and 6-8 candidates per batch. By March 2008, about 34 Medical Officers had been trained for comprehensive EmOC and posted in well-equipped centres. About 40 staff nurses have been trained to help these service providers.

### Training of MBBS doctors on anaesthesia

There is a dearth of qualified anaesthetists in the public sector of rural Gujarat. The Government of Gujarat started training general doctors for obstetric anaesthesia in four teaching hospitals to meet the demand following the Government of India guidelines. A certificate is given to them at the end of 18-week training by the State Institute of Health and Family Welfare after conducting oral and written examinations. The doctors are posted in areas needing specialists and equipped for surgeries. Forty-four doctors have so far been trained on anaesthesia, and 16 are currently in training. Unfortunately the anaesthesia society is not involved in this training and is somewhat opposed to it.

### Training of skilled birth attendants for normal deliveries

The Government of India started an initiative to train ANMs for skilled birth attendance in 2005. Gujarat is one of the initial states to start the training at selected centres based on the Government of India's SBA guidelines; trained ANMs are provided with equipment and supplies. This 15-day competency-based hands-on training also uses models and techniques for adult learning. Till March 2008, about 4,500 of almost 7,500 ANMs have been trained. Unfortunately, there is no systematic MIS to record if these trained ANMs have been providing more or better maternal care.

### Innovations in recruitment and initial posting policy

Given the chronic shortage of medical officers and specialists, the Government of Gujarat has developed a unique way of recruiting doctors and paramedical staff, called ‘walk-in recruitment'. In this system, candidates walk into the RDD's office with the certificates and are given an appointment letter on the spot if the criteria of appointment are fulfilled. The candidate is given a posting at a place of his/her choice from available vacant positions. This cuts short the typical delays in initial posting of staff.

### Verbal autopsy for maternal death audit

The Government of Gujarat has begun registration of maternal deaths and verbal autopsies since September 2006. Initial forms were revised based on feedback from the field, and a software was developed for data-entry and analysis. The forms are distributed to all block health officers who conduct verbal autopsies based on information of maternal death from field workers. The forms are sent to the district level, and data are entered using the software. The soft copy is then sent to the state level where compilation and analysis of data are done. Since it is a new system, there are issues of under-reporting of deaths, improper filling up of forms, and absence of feedback from higher levels. Little analysis has been done on the collected data, and no report has yet been published. On the positive side, there is an increase in awareness regarding the tragedy of maternal deaths among the community and field workers because of the registration of maternal deaths and verbal autopsy. State- and district-level officers are also realizing various socioeconomic issues and gaps in the public-health system which lead to maternal deaths. Unfortunately, registration of maternal deaths and verbal autopsy are not being systematically followed up because no one is singularly responsible at the district, region, or state level for this activity. This is in contrast to the system in Tamil Nadu where the Collector (the highest government employee) conducts the maternal death inquiry in each district, and data are compiled and closely monitored by the Director of Public Health and Commissioner, MCH.

## CONCLUSION

The Gujarat state of India has come a long way in improving the health indicators since independence of the Subcontinent. However, progress in reducing maternal mortality has been slow and largely unmeasured and undocumented. Lack of skilled staff, inadequate infrastructure, and poor monitoring have led to the under-use of the public-health system for delivery care.

Recent innovations in maternal health in Gujarat have been driven by a dynamic, health Commissioner trained in public health and with long experience in the health sector. He has increased the technical management capacity in the Commissionerate by appointing a couple of selected officers with public-health and obstetrician and gynaecologist background in charge of EmOC and training and hired young health-management graduates for improving programme management at the district level. The Commissioner has also involved the private sector, management training institutions, and NGOs for advice and design of innovations.

The public-health system in Gujarat faces the same set of problems relating to infrastructure, human resources, and management as the rest of the country. Despite the laudable new initiatives, such as the Chiranjeevi Yojana, training of MBBS doctors, and other innovations to fill the staff vacancies, the absence of a reliable system for the registration of maternal deaths makes it difficult to measure progress.

## RECOMMENDATIONS

Following are some recommendations to improve maternal health services in Gujarat:

- Management capacity should be improved by creating additional permanent posts for maternal health at the directorate to plan, implement, and monitor safe motherhood programmes in Gujarat. In addition, officers at the directorate, including those at the region and district levels, should have training on management and public-health skills.
- Focus must be on ensuring that all the FRUs are made fully functional with comprehensive EmOC services, including blood, and at least 50% of the PHCs and all the CHCs provide delivery care, including basic EmOC services, on a 24/7 basis. Progress towards this goal must be annually monitored.
- Gujarat needs to establish a reliable vital registration system, especially for maternal and neonatal mortality and a maternal outcome-monitoring system to have an accurate picture of maternal health situation. This will also help assess the impact of interventions on maternal mortality.
- A cadre of midwives should be developed to make skilled delivery care available to all women. Posts of an institutional and a community-based midwife should be created in both government and private facilities to look after normal deliveries and basic EmOC. A facility-based midwifery model needs to be developed to provide round-the-clock delivery care with a specialist back-up to provide comprehensive maternal healthcare.
- Training general doctors for comprehensive EmOC, including caesarean section and anaesthesia, will help improve access to comprehensive EmOC. The trained doctors should be supported by adequate infrastructure, regular supplies, and legal protection. Technical backstopping and periodic supervision by trainers and obstetricians and gynaecologists is important to give them confidence to carry out operative procedures and managing complicated cases.
- Improvements and maintenance of the health infrastructure of the existing infrastructure are necessary to provide good quality maternal health services.
- Blood is essential to save a mother if she has a haemorrhage, especially for anaemic mothers. Gujarat needs to improve access to blood for obstetric cases. Scaling up establishment of blood-storage centres at the subdistrict level will help improve access to blood. Training of general doctors for blood-banking services will remove the human resource barrier for blood-banking services.

With new initiatives, such as training of doctors for specialized skills and public-private partnerships for improving maternal health services, Gujarat is one of the progressive states in India. The goal of reducing maternal mortality to less than 100 by 2010 can be achieved only by creating awareness among policy-makers, implementing evidence-based interventions for reduction in maternal mortality, and through mobilization of professionals and society. The speed of reduction in maternal mortality will depend on strategies adopted to ensure skilled care at all births backed up by EmOC, the extent of accountability of professionals responsible for maternal health, and the willingness of politicians to support maternal health programmes by allocating resources to strengthen the health system.

## ACKNOWLEDGEMENTS

This publication was supported by a fund provided by the Department for International Development (DFID), UK. Its contents are solely the responsibility of the authors and do not necessarily represent the official views of DFID.
